# Assessing the performance of large language models (GPT-3.5 and GPT-4) and accurate clinical information for pediatric nephrology

**DOI:** 10.1007/s00467-025-06723-3

**Published:** 2025-03-05

**Authors:** Nadide Melike Sav

**Affiliations:** Department of Pediatric Nephrology, Duzce University, Duzce, Turkey

**Keywords:** Artificial intelligence, ChatGPT, Clinical decision support systems, Cohen’s *d*, Cronbach’s alpha, Pediatric nephrology

## Abstract

**Background:**

Artificial intelligence (AI) has emerged as a transformative tool in healthcare, offering significant advancements in providing accurate clinical information. However, the performance and applicability of AI models in specialized fields such as pediatric nephrology remain underexplored. This study is aimed at evaluating the ability of two AI-based language models, GPT-3.5 and GPT-4, to provide accurate and reliable clinical information in pediatric nephrology. The models were evaluated on four criteria: accuracy, scope, patient friendliness, and clinical applicability.

**Methods:**

Forty pediatric nephrology specialists with ≥ 5 years of experience rated GPT-3.5 and GPT-4 responses to 10 clinical questions using a 1–5 scale via Google Forms. Ethical approval was obtained, and informed consent was secured from all participants.

**Results:**

Both GPT-3.5 and GPT-4 demonstrated comparable performance across all criteria, with no statistically significant differences observed (*p* > 0.05). GPT-4 exhibited slightly higher mean scores in all parameters, but the differences were negligible (Cohen’s *d* < 0.1 for all criteria). Reliability analysis revealed low internal consistency for both models (Cronbach’s alpha ranged between 0.019 and 0.162). Correlation analysis indicated no significant relationship between participants’ years of professional experience and their evaluations of GPT-3.5 (correlation coefficients ranged from − 0.026 to 0.074).

**Conclusions:**

While GPT-3.5 and GPT-4 provided a foundational level of clinical information support, neither model exhibited superior performance in addressing the unique challenges of pediatric nephrology. The findings highlight the need for domain-specific training and integration of updated clinical guidelines to enhance the applicability and reliability of AI models in specialized fields. This study underscores the potential of AI in pediatric nephrology while emphasizing the importance of human oversight and the need for further refinements in AI applications.

**Graphical abstract:**

**Figure:**
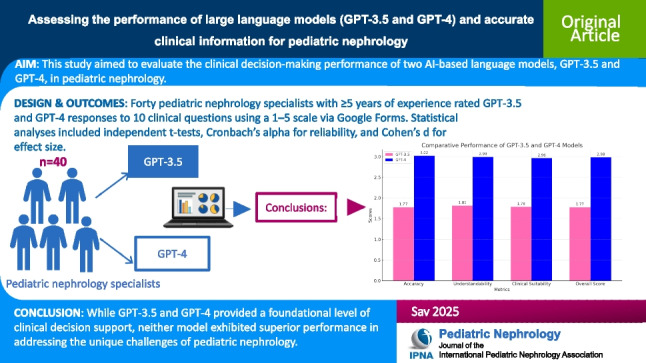
A higher resolution version of the Graphical abstract is available as Supplementary information

**Supplementary Information:**

The online version contains supplementary material available at 10.1007/s00467-025-06723-3.

## Introduction

In recent years, the rapid integration of artificial intelligence (AI) technologies into healthcare has significantly advanced clinical decision support systems and other applications [1, 2]. One of the most prominent advantages of AI lies in its ability to process vast amounts of data, thereby assisting healthcare professionals in making faster and more informed decisions. Natural language processing (NLP) technologies, in particular, have revolutionized the interpretation and processing of text-based information in medicine [3, 4]. Large language models, such as ChatGPT, have the potential to provide quick and comprehensible answers to complex clinical queries, potentially bridging gaps in medical knowledge and practice [5, 6].

Pediatric nephrology is a highly specialized field that involves the early diagnosis, accurate treatment, and long-term management of various kidney disorders in children. The complexity of pediatric kidney diseases requires precise knowledge and multidisciplinary approaches. Thus, there is a critical need for innovative technologies that can offer reliable and accurate information to support healthcare professionals. However, studies assessing the application and effectiveness of AI models in specific specialties, such as pediatric nephrology, remain scarce [7].

Existing literature highlights the potential utility of AI-based systems in several medical specialties. For instance, studies in ophthalmology, cardiology, and dermatology have demonstrated that AI technologies can enhance diagnostic accuracy and expedite accurate clinical information processes [8–10]. However, the performance of these models in addressing the unique challenges of pediatric nephrology has not been adequately explored [7, 11]. This lack of evidence underscores the importance of conducting focused evaluations to understand the applicability and reliability of AI tools in this domain.

The primary objective of this study is to evaluate the accurate clinical information capabilities of ChatGPT’s GPT-3.5 and GPT-4 models in the context of pediatric nephrology. Specifically, the study aims to assess the models’ ability to provide accurate, comprehensive, and patient-friendly responses to clinical questions commonly encountered in pediatric nephrology practice. The responses were analyzed based on four key criteria: accuracy, scope, comprehensibility, and clinical applicability. Furthermore, the study seeks to identify any comparative advantages or limitations between the two models.

This research represents one of the first comparative evaluations of AI-based systems in the field of pediatric nephrology. The findings are expected to contribute not only to improving clinical practices within this specialty but also to guiding the adoption of AI technologies in other medical disciplines. By addressing a significant gap in the literature, this study is aimed at providing valuable insights into the practical integration of AI in specialized healthcare settings.

## Methods

### Study design and participants

This observational and comparative study aimed to evaluate the performance of artificial intelligence-based language models (GPT-3.5 and GPT-4) in providing accurate clinical information for pediatric nephrology. Forty pediatric nephrology specialists with at least 5 years of professional experience voluntarily participated in the study. Participants included academic specialists, clinicians, and those with combined academic and clinical roles.

The study adhered to the principles of the Declaration of Helsinki and was approved by the Duzce University Ethics Committee (approval number 2024/218). Participants were informed about the study’s objectives, procedures, potential risks, and benefits before providing written consent. To minimize bias and ensure confidentiality, all responses were anonymized and analyzed independently.

This study was conducted in accordance with the STROBE (Strengthening the Reporting of Observational Studies in Epidemiology) guidelines, ensuring transparency, reproducibility, and completeness in reporting. All data were used solely for research purposes and were handled in a manner that safeguarded participant confidentiality.

### Data collection method

Data were collected using an online survey hosted on Google Forms. Participants were informed about the study’s objectives, procedures, and their rights before completing the survey. The survey included responses generated by GPT-3.5 and GPT-4 for 10 clinical questions commonly encountered in pediatric nephrology. Participants evaluated the responses on a 5-point Likert scale, where 1 indicated insufficient performance and 5 indicated very sufficient performance. The evaluation focused on four criteria: accuracy, defined as the correctness and reliability of the information; scope, reflecting the comprehensiveness of the response; patient friendliness, assessing the clarity and accessibility of the response for patients and caregivers; and practical applicability, evaluating the relevance and usefulness of the information in the provision of accurate clinical information.

### Development of clinical questions

The clinical questions were developed using a systematic and data-driven approach. Anonymized data from the hospital’s information technology department were analyzed to identify the most common clinical scenarios managed by the pediatric nephrology department over the past 10 years. These scenarios included conditions such as nephrotic syndrome, acute kidney injury, chronic kidney disease, electrolyte imbalances, hypertension, dialysis, and kidney transplantation. Based on this analysis, 10 clinical questions were formulated to reflect real-world challenges in pediatric nephrology.

The draft questions were reviewed and validated by three senior pediatric nephrologists to ensure their alignment with current clinical guidelines and their relevance to everyday clinical practice. The finalized questions encompassed diagnostic, therapeutic, and management aspects of pediatric nephrology, ensuring a comprehensive evaluation of the AI models’ ability to provide accurate clinical information (see Table 1).

**Table 1:** Clinical questions used in the evaluation

Question number	Clinical question
1	What are the early signs of chronic kidney disease in children, and which tests are used for diagnosis?
2	What factors should be considered when selecting antibiotics for treating a child with a urinary tract infection?
3	What is the most common cause of nephrotic syndrome in children, and what is the first-line treatment?
4	What are the emergency diagnostic and treatment steps for a child with acute kidney failure?
5	How should a long-term management plan be created for a child diagnosed with acute glomerulonephritis?
6	What drugs and approaches are preferred in the treatment of hyperkalemia in children?
7	How is the prognosis of a patient diagnosed with autosomal dominant polycystic kidney disease assessed?
8	How should dietary management be planned for a child starting dialysis, and what factors should be considered?
9	What are the most common causes of hypertension in children, and how should the diagnostic process be conducted?
10	What are the major complications encountered during immunosuppressive therapy follow-up after transplantation?

### Statistical analysis

Statistical analyses were performed using SPSS version 22.0. Descriptive statistics, including means, standard deviations, frequencies, and percentages, were calculated to summarize the demographic characteristics of the participants and their evaluations of the AI models. Independent *t*-tests were used to compare the mean scores of GPT-3.5 and GPT-4 across the four evaluation criteria: accuracy, scope, patient friendliness, and practical applicability [12]. A *p*-value of less than 0.05 was considered statistically significant [13].

Reliability analysis was conducted using Cronbach’s alpha to assess the internal consistency of participants’ ratings for each model and criterion [14]. Effect sizes were calculated using Cohen’s *d* to quantify the magnitude of differences between the two models [15]. Additionally, Pearson correlation analysis was used to examine the relationship between participants’ years of professional experience and their evaluation scores for GPT-3.5 [16]. All statistical analyses were conducted in accordance with standard practices to ensure the robustness of the results [17].

## Results

This study was conducted with 40 experienced pediatric nephrology physicians. Participants had a minimum of 5 years of professional experience and represented diverse academic and clinical roles. The professional experience of participants varied according to their respective roles. Table 2 provides demographic information and professional experience categorized by participant roles.

**Table 2:** Demographic information and professional experience of participants by category

Category	Count *n*	Percentage %	Average years of experience
Academics	14	35.0	7.5 years
Both clinician and academic	13	32.5	8.2 years
Clinicians	13	32.5	9.3 years

The comparative analysis of GPT-3.5 and GPT-4 was conducted based on their responses to clinical questions regarding pediatric nephrology. The models were evaluated on four key criteria: accuracy, scope, usability, and comprehensibility. The findings revealed similar performance between the two models, as shown in Table 3, which includes reliability (Cronbach’s alpha) and effect size (Cohen’s *d*) values.

**Table 3:** Reliability values and effect sizes of the models

Criterion	Cronbach’s alpha (GPT-3.5)	Cronbach’s alpha (GPT-4.0)	Cohen’s *d* (3.5 vs. 4.0)
Accuracy	0.019	0.162	− 0.011
Scope	0.019	0.162	0.010
Usability	0.019	0.162	0.040
Comprehensibility	0.019	0.162	0.065

The *p*-values obtained from statistical tests indicated no significant differences between the models’ performances across all criteria (Table 4). Both models demonstrated comparable levels of accurate clinical information support.

**Table 4:** *p*-values for the comparison of two AI models

Criterion	*p*-value
Accuracy	0.879
Scope	0.882
Usability	0.568
Comprehensibility	0.356

Additionally, correlation analyses between years of professional experience and GPT-3.5 scores on the four evaluation criteria were conducted. These analyses revealed no significant correlations, suggesting that professional experience did not substantially influence model evaluations. The correlation coefficients are presented in Table 5.

**Table 5:** Correlation coefficients between professional experience and GPT-3.5 scores

Criterion	Correlation coefficient
Accuracy	0.037
Scope	− 0.026
Usability	0.048
Comprehensibility	0.074

**Fig. 1:**
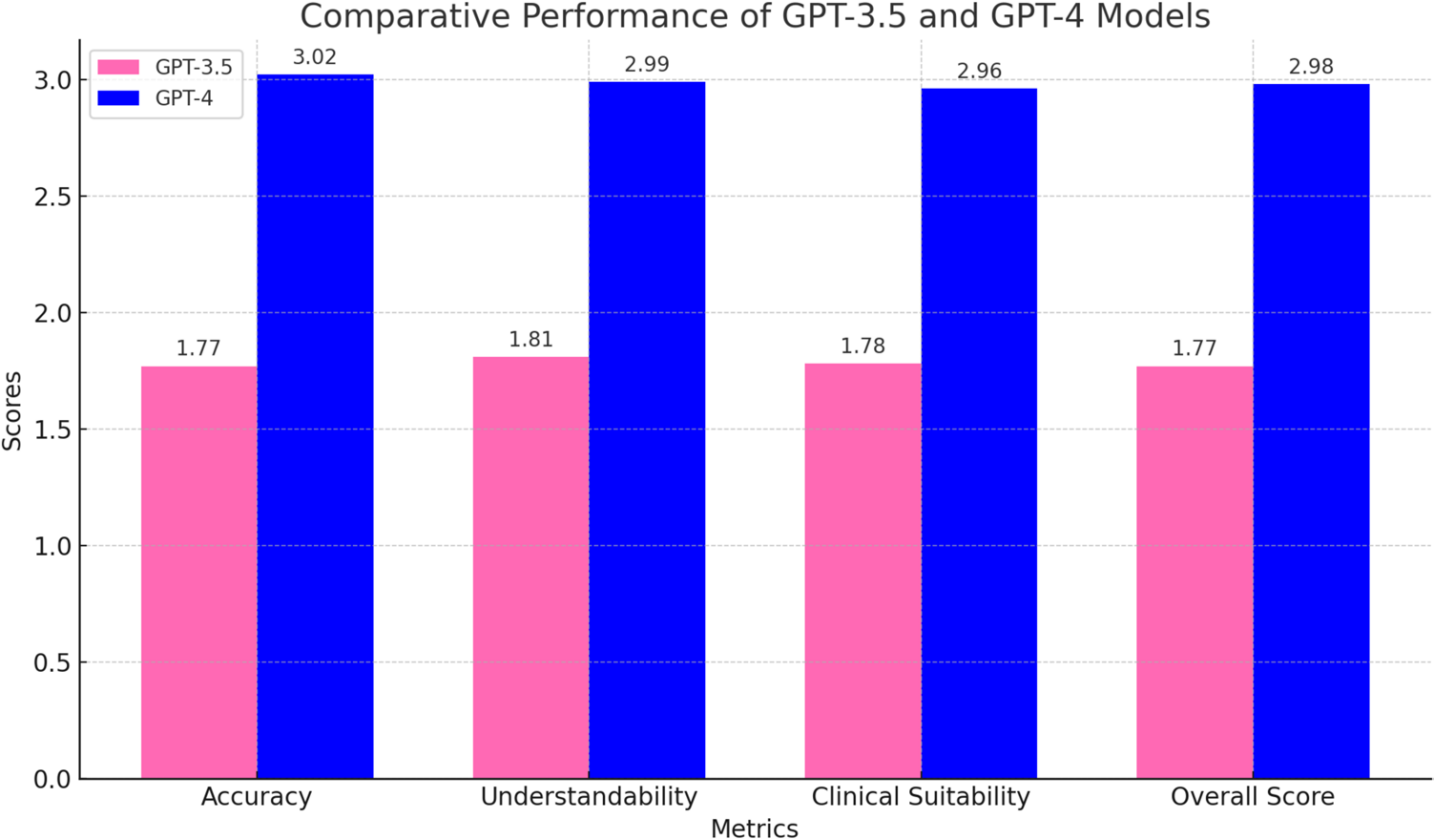
An illustration of the average scores for GPT-3.5 (pink) and GPT-4 (blue) on four accurate clinical information criteria: accuracy, comprehensibility, clinical suitability, and overall score. While GPT-4 outperformed GPT-3.5 in all metrics, the differences were not statistically significant, as confirmed by the p-values in Table 3

Finally, Fig. 1 visually compares the performance of GPT-3.5 and GPT-4 across the four accurate clinical information criteria. The scores indicated that while GPT-4 consistently achieved higher scores than GPT-3.5, the differences were not statistically significant (See the Supplementary Material for GPT-3.5 and GPT-4 questions and answers).

## Discussion

This study provides a comprehensive evaluation of the accurate clinical information capabilities of AI-based language models, GPT-3.5 and GPT-4, in pediatric nephrology using four key parameters: accuracy, scope, patient-friendly comprehensibility, and clinical applicability. The findings indicated that both models performed similarly across these parameters, with no statistically significant differences observed. These results, when contextualized within the existing literature, offer important insights and highlight areas for further improvement and development [7, 18].

Accuracy is a critical parameter for AI models in clinical applications, as accurate diagnostic and treatment suggestions can significantly enhance decision-making processes for healthcare professionals. In this study, GPT-4 showed a slight tendency to provide more accurate responses than GPT-3.5, but the difference was not statistically significant, suggesting that both models possess similar levels of medical knowledge [19, 20].

In the literature, studies have demonstrated that GPT-4 often outperforms GPT-3.5 in terms of accuracy across various medical specialties [21, 22]. For instance, Yudovich et al. (2024) reported that GPT-4 performed better on standardized urology knowledge assessments compared to GPT-3.5 [23]. Similarly, Jo et al. (2024) noted that GPT-4 demonstrated higher accuracy in text-based outpatient recommendations [24]. However, the domain of pediatric nephrology, being a more specialized and complex field, may require training data that are both highly specific and up-to-date to improve accuracy further. Integrating current guidelines and domain-specific literature into training datasets could significantly enhance the models’ accuracy in this context.

The scope parameter measures the depth and breadth with which a model addresses a given question. In this study, GPT-4 marginally outperformed GPT-3.5 in terms of scope, suggesting a broader knowledge base. However, the difference was not statistically significant, indicating comparable performance between the two models.

The scope of AI models is highly dependent on the quality, diversity, and recency of the training data. Bahir et al. (2024) demonstrated that ChatGPT provided detailed and clinically useful answers to patient-centered questions in ophthalmology [25]. However, in highly specialized domains like pediatric nephrology, the models’ limited access to domain-specific datasets might restrict their scope. Enhancing the breadth and depth of training datasets specific to pediatric nephrology could help the models provide more comprehensive and nuanced responses.

Patient-friendly comprehensibility refers to the ability of AI models to simplify complex medical information into language that patients or their caregivers can easily understand. In this study, GPT-4 demonstrated slightly better performance in this regard, but the difference was not statistically significant. Both models were found to be generally capable of providing comprehensible responses.

The literature suggested that large language models are increasingly effective at simplifying medical jargon for non-expert audiences [26]. Nazi et al. (2024) highlighted that GPT-4 provided clearer and more comprehensible responses in text-based patient guidance compared to earlier models [27]. However, in pediatric nephrology, where precise communication with caregivers is crucial, the balance between simplification and preservation of critical information is vital. Over-simplification might lead to loss of essential details, which could impact accurate clinical information or patient management.

Clinical applicability reflects the practical utility and reliability of AI models in real-world clinical settings. In this study, both models showed similar performance in this parameter, with no statistically significant difference. This finding aligned with prior research emphasizing the need for cautious integration of AI models into clinical workflows. One critical aspect of GPT models, such as GPT-3.5 and GPT-4, is their potential to produce hallucinations or confidently presented but incorrect information. In clinical settings, this challenge underscores the importance of ensuring the accuracy and reliability of AI-generated outputs, particularly in fields like pediatric nephrology, where complex cases demand precise and evidence-based guidance. These inaccuracies highlight the need for healthcare professionals to validate and monitor AI recommendations, ensuring they align with current clinical standards. Moreover, refining the training datasets to incorporate domain-specific knowledge and updated guidelines is essential for reducing the occurrence of hallucinations. As the technology evolves, GPT models can serve as valuable complementary tools to enhance clinical workflows, provided they are used in conjunction with human expertise and oversight. The integration of artificial intelligence into clinical practice raises significant ethical considerations, particularly regarding accountability, bias, and patient safety. AI models, including GPT-3.5 and GPT-4, operate based on training data that may inherently reflect societal biases, potentially influencing the recommendations or conclusions provided. In pediatric nephrology, such biases could disproportionately impact certain patient groups, leading to disparities in care. Additionally, the “black box” nature of many AI algorithms makes it challenging for clinicians to interpret and trust AI-generated outputs, raising questions about accountability in cases where AI suggestions contribute to adverse patient outcomes. Ensuring the ethical use of AI requires rigorous oversight, including continuous validation of AI outputs against current clinical guidelines and standards, transparency in AI model development, and comprehensive training for clinicians on AI limitations. As these technologies continue to evolve, a multidisciplinary approach involving ethicists, healthcare providers, and AI developers will be essential to address these challenges and ensure that AI tools are implemented in a manner that prioritizes patient welfare and equity.

Jo et al. (2024) emphasized that while AI models could provide valuable information, their recommendations should be interpreted as supportive rather than definitive, particularly in complex medical fields [24]. Bekbolatova et al. (2024) also underscored the potential risks of relying solely on AI models for accurate clinical information, advocating instead for their use as tools to complement human expertise [28]. These findings suggest that while AI models like GPT-3.5 and GPT-4 hold promise, they are not yet ready to independently guide clinical practice in specialized fields such as pediatric nephrology. Integrating domain-specific training and ensuring alignment with clinical guidelines will be critical for enhancing their clinical applicability.

The findings of this study align with broader trends in the literature on AI in medicine [29, 30]. Previous research has consistently shown that AI models perform well in general medical domains but face challenges in specialized areas where domain-specific knowledge is critical [31]. For instance, Yudovich et al. (2024) found that GPT-4 demonstrated strong performance in urology but emphasized its reliance on pre-existing training data [23]. Similarly, Balci et al. (2024) reported that while ChatGPT provided clinically useful responses in ophthalmology, its performance varied depending on the complexity and specificity of the clinical scenarios [32].

These observations reinforce the need for targeted efforts to train AI models using data tailored to specialized medical fields. By incorporating updated pediatric nephrology guidelines, case studies, and real-world clinical scenarios into training datasets, the performance of AI models could be further optimized, ensuring greater reliability and utility in clinical practice.

This study has several limitations. First, the sample size of 40 pediatric nephrology experts may limit the generalizability of the findings. Larger studies involving diverse participants from multiple centers could provide more robust and representative data. Second, the clinical questions used in this study, though carefully designed, were limited to 10 scenarios and may not fully capture the complexity and variability of pediatric nephrology practice. Expanding the scope of clinical scenarios to include more specific and nuanced cases—such as real-world patient cases rather than generalized theoretical questions—could yield deeper insights into the performance of AI models in this specialized field. This methodological choice was made to ensure a standardized and reproducible evaluation process; however, future studies incorporating patient-specific cases may provide a more clinically relevant assessment of AI performance.

Third, the subjective nature of the scoring system introduces variability based on individual perspectives and experiences. While subjective evaluations offer valuable insights, incorporating objective metrics or standardized frameworks in future studies could improve the reliability and reproducibility of assessments. Additionally, the risk of selection bias due to voluntary participation must be acknowledged, as this could influence the representativeness of participant feedback.

Fourth, a significant limitation of GPT models like GPT-3.5 and GPT-4 is their tendency to produce “hallucinations” or confidently stated but incorrect information. This issue poses a critical challenge in clinical contexts, where accuracy and reliability are paramount. In pediatric nephrology, where decisions often involve complex and sensitive cases, hallucinations could lead to inappropriate recommendations if not carefully monitored. This highlights the need for continuous oversight by healthcare professionals and improvements in the training datasets to reduce such risks.

Fifth, the observed similarity in performance between GPT-3.5 and GPT-4 in this study differs from findings in other domains where GPT-4 generally demonstrates superior accuracy. This could be attributed to the nature of the questions used, which focused on fundamental clinical knowledge rather than complex reasoning or multi-step problem-solving. Future research should explore AI performance in scenarios that require deeper clinical judgment and decision-making to better capture potential performance differences between model versions.

Finally, this study focused exclusively on GPT-3.5 and GPT-4, without including other AI models or comparative systems. Exploring the capabilities and limitations of a broader range of AI technologies, including those specifically designed for medical applications, could provide a more comprehensive understanding of how AI can be integrated into clinical workflows.

## Conclusion

This study demonstrates that GPT-3.5 and GPT-4 exhibit comparable performance in supporting accurate clinical information processes in pediatric nephrology across four key parameters: accuracy, scope, comprehensibility, and clinical applicability. While both models offer a foundational level of clinical decision support, their performance could be significantly enhanced through the inclusion of more domain-specific and updated training data.

In pediatric nephrology, a highly specialized and sensitive field, AI models are not yet positioned to independently guide clinical practice. However, their potential as complementary tools for healthcare professionals is evident. Future research should focus on integrating these technologies with clinical guidelines and expanding their training datasets to ensure their effective and reliable application in specialized medical fields. This study serves as an important step toward understanding the capabilities and limitations of AI in pediatric nephrology and provides a foundation for future advancements in this domain.

## Supplementary Information

Below is the link to the electronic supplementary material.

Graphical abstract (PPTX 175 KB)

## Data Availability

All data generated or analyzed during this study are included in this published article (and its supplementary information files).
