# Supplementary material for: Assessing the performance of large language models (GPT-3.5 and GPT-4) and accurate clinical information for pediatric nephrology
Source: Pediatr Nephrol. 2025 Mar 5;40(9):2879–85. doi: 10.1007/s00467-025-06723-3 (PMC12296824; doi:10.1007/s00467-025-06723-3)
Supplement: Supplementary file 1 — Graphical abstract (PPTX 175 KB) [file 467_2025_6723_MOESM1_ESM.pptx]

## Slide 1
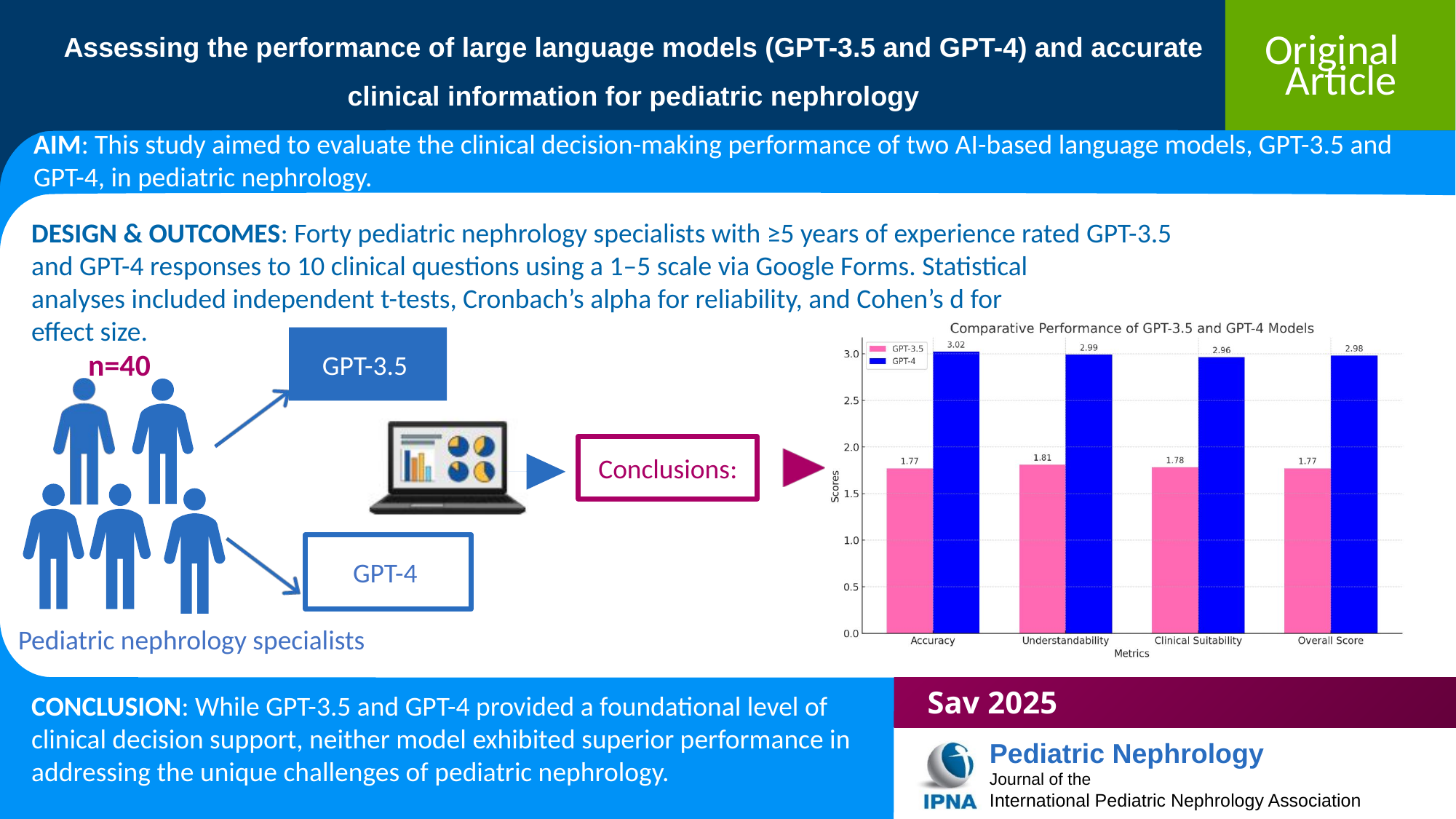

Assessing the performance of large language models (GPT-3.5 and GPT-4) and accurate clinical information for pediatric nephrology
AIM: This study aimed to evaluate the clinical decision-making performance of two AI-based language models, GPT-3.5 and GPT-4, in pediatric nephrology.
DESIGN & OUTCOMES: Forty pediatric nephrology specialists with ≥5 years of experience rated GPT-3.5
and GPT-4 responses to 10 clinical questions using a 1–5 scale via Google Forms. Statistical
analyses included independent t-tests, Cronbach’s alpha for reliability, and Cohen’s d for
effect size.
GPT-3.5
n=40
Conclusions:
GPT-4
Pediatric nephrology specialists
Sav 2025
CONCLUSION: While GPT-3.5 and GPT-4 provided a foundational level of clinical decision support, neither model exhibited superior performance in addressing the unique challenges of pediatric nephrology.
